# Supplementary material for: Correction: Optimizing large-scale gambling prevention with adolescents through the development and evaluation of a training course for health professionals: The case of PRIZE
Source: PLoS One. 2024 Dec 5;19(12):e0315431. doi: 10.1371/journal.pone.0315431 (PMC11620382; doi:10.1371/journal.pone.0315431)
Supplement: S2 File — (PDF) [file pone.0315431.s002.pdf]

RESEARCH ARTICLE

# Optimizing large-scale gambling prevention with adolescents through the development and evaluation of a training course for health professionals: The case of *PRIZE*

**Maria Anna Donati[1]*, Jessica Boncompagni[1], Giuseppe Iraci Sareri[2], Sonia Ridolfi[2], Adriana Iozzi[3], Valentina Cocci[4], Alfiero Arena[5], Caterina Primi[1]**

**1** NEUROFARBA Department – Section of Psychology, University of Florence, Florence, Italy, **2** CEART (Coordinamento Enti Ausiliari Regione Toscana), Florence, Italy, **3** UFC SerD Zona 1 Firenze, Azienda USL Toscana Centro, Florence, Italy, **4** UFC SerD Arezzo, Azienda USL Toscana Sud Est, Florence, Italy, **5** UF SerD Valle del Serchio, Azienda USL Toscana Nord Ovest, Florence, Italy

* mariaanna.donati@unifi.it

Check for updates

OPEN ACCESS

**Citation:** Donati MA, Boncompagni J, Iraci Sareri G, Ridolfi S, Iozzi A, Cocci V, et al. (2022) Optimizing large-scale gambling prevention with adolescents through the development and evaluation of a training course for health professionals: The case of *PRIZE*. PLoS ONE 17(5): e0266825. https://doi.org/10.1371/journal.pone.0266825

**Editor:** Marc Potenza, Yale University, UNITED STATES

**Received:** December 12, 2021

**Accepted:** March 28, 2022

**Published:** May 12, 2022

**Copyright:** © 2022 Donati et al. This is an open access article distributed under the terms of the Creative Commons Attribution License, which permits unrestricted use, distribution, and reproduction in any medium, provided the original author and source are credited.

**Data Availability Statement:** All relevant data are within the manuscript and its Supporting Information files.

**Funding:** The program was funded by Tuscany Region (Resolution of the Tuscany Region n. 771, 9 July 2018) through ANCI TOSCANA (Association of Tuscan Municipalities).

**Competing interests:** The authors have declared that no competing interests exist

## Abstract

In the field of adolescent gambling prevention, there is a lack of intervention studies reporting and assessing training courses for the intervention providers. The present work fills this gap by realizing a dissemination study inside the PRIZE program aimed at modifying a set of cognitive protective factors and affective risk factors. The purpose of this work was twofold: To develop and evaluate a training course with the intervention providers (Study 1), and to assess the short- and long-term effects of the intervention itself (Study 2). The training course was delivered to 44 health professionals (32 females, $M_{age}$ = 39.34 years). Results showed a significant increase of correct knowledge about gambling and a significant reduction of their susceptibility to probabilistic reasoning biases. Participants also actually learnt the main competencies to conduct the educational activities, they were satisfied for the training course received, and they felt high levels of self-efficacy. The intervention was implemented with 1894 high school students (61% males; $M_{age}$ = 15.68 years). In the short term, we found a significant increase of adolescents' correct gambling knowledge, random events knowledge, and probabilistic reasoning ability, and a significant decrease of superstitious thinking, monetary positive outcome expectation, and gambling-related erroneous thoughts and fallacious behavioral choices. In the long-term, a significant decrease of gambling frequency, gambling versatility, and gambling problem severity was obtained. Overall, this work highlights the importance to train prevention program providers in order to optimize the effectiveness of large-scale gambling intervention programs towards adolescents.

## Introduction

One of the increasingly widespread risky behaviour for health in adolescence is gambling, that, if practiced excessively, can evolve into at-risk or problematic behaviour, intended as an excessive behaviour that causes negative consequences for the gambler's life, for his/her social

context, or for community [1]. Numerous studies have provided estimates of involvement in gambling and of risky/problem behaviours associated with it in adolescents [2] attesting that adolescent problem gambling is a public health problem. Calado and colleagues [3], considering 44 studies conducted in different contexts (Europe, Asia, Oceania, North and South America), found that 0.2-12.3% of adolescents showed problematic gambling behaviours. Concerning Italy, in which the amount wagered in the gambling market from 2015 to 2019 has increased from 88.2 to 110.54 billion euros [4], gambling prevalence among adolescents is estimated to be 32% in 2019 considering the last 12 months, with Italy that is the third European country for gambling among youth [5], despite the law banning gambling for minors. More recent data indicate a higher prevalence of gambling, with 75% of young past-year gamblers, engaging mostly on scratch-cards (52%), bingo (38%) and sports bets (35%) [6]. Moreover, a survey carried out by the Italian Institute of Health in 2017 evidenced that 6.5% of students (14-17 years old) were at-risk or showed problems related to gambling [7].

On the light of the statistics, it is imperative to develop and implement preventive actions to reduce the risk for gambling problems in youth, particularly in Italy. Several educational and preventive programs have been designed, realized and evaluated to prevent problem gambling in youth [8–10]. Nevertheless, some points of weakness have to be recognized. Among these, there is the general lack of attention on developing the educational activities based on evidence-based theoretical models [8] and this this may be responsible for insufficient evidence of actual change in gambling behaviour as an effect of the interventions [8]. Moreover, various different professional figures to implement the educational intervention in the school contexts are usually employed [11–15]. However, although clear guidelines about the desirable specific figures to do the intervention still do not exist, it has been demonstrated that gambling specialists are more effective than school teachers in reducing gambling-related misconceptions [9]. Strictly related to this aspect, there is the general lack of training courses for the health professional figures involved in the implementation of the educational activities with the juveniles, and, consequently, the verification of their efficacy is missing. In particular, from the literature analysis, it emerges that the articles reporting gambling prevention programs do not specify whether these figures have been trained or, in the cases where they were trained, it is not described how, and if the training course has been effective in reaching the planned aims [15, 16].

This represents a very important problem as one of the factors that affect the efficacy of preventive interventions in the health domain is the good quality of the training addressed to the people who will implement the programs [17]. Indeed, trainings allow those who carry out the program to become confident with it [18]. Moreover, some interventions need certain competencies which can be acquired only with specific training courses [19], and a lack of training can be considered as an obstacle to provide some prevention actions [20]. Recruiting, selecting, training, and supporting interpersonally skilled, high-quality staff are essential to beneficial programming, especially in interventions addressed to the juveniles. In particular, a well-planned staff development provides basic theoretical knowledge, clear program goals and objectives, modeling and practice of effective intervention strategies, regular coaching, and constructive feedback [21]. Prevention programming must be effectively implemented to produce optimal outcomes as the program's impact is mediated by the program providers' personal efficacy, mastery in conveying program content, warmth, empathy, humor, relationship skills, and capacity to guide and foster the skill development and application of young people [22]. Thus, it is important assessing the efficacy of these trainings through appropriate studies in order to analyze the factors that affect the quality of the training itself.

Following these premises, the general goal of this work was to realize and evaluate a gambling prevention intervention for adolescents that fills the weaknesses characterizing previous intervention studies [8]. In detail, we aimed at implementing a large-scale a school-based

universal intervention, i.e., *PRIZE* [Prevention of gambling risks among adolescents], in the school year 2019-2020, after having designed, realized, and evaluated a training course for the intervention providers. We implemented the intervention in Tuscany, where , it has been found that 11% of young gamblers were at risk gamblers and 8% were problem gamblers [6].

As recommended by the guidelines published by the Society for Prevention Research for the development of effective preventive interventions [23], PRIZE was based on an empirically evaluated explanation and intervention model [11]. This model employed the dual process theory on cognitive functioning to explain the development of gambling problems in adolescents [24], consistent with what previously evidenced with adults [25]. The dual-process theory distinguishes between autonomous sets of systems (rapid, automatic, parallel, and heuristic) and analytic cognitive processes (slow, under control, serial, and rule-based) [24]. Donati et al. [11] used the dual-process model to explain how adolescents tend to have high levels of cognitive distortions about gambling, i.e., a wide array of mistaken beliefs and irrational perceptions about gambling [26], and, consequently, to be more prone to gamble frequently and to develop gambling problems [27]. Particularly, they took into account the concept of *mindware* [28], that can be defined as the rules, procedures, and strategies derived from past learning experiences and available for explicit retrieval. As antecedents of reasoning failures, there can be a *mindware gap* or a *contaminated mindware*. There is a *mindware gap* when the appropriate rules, procedures, and strategies are lacking, while a *contaminated mindware* verifies when the employed mindware is not helpful in the specific situation. In detail, a *mindware gap* there can be when there is a misunderstanding or randomness [14] or a lack of knowledge concerning probability [29]. In other words, a *mindware gap* can occur when there is a shortage of *cognitive protective factors*, as randomness and probabilistic knowledge, competencies, and ability to resist to fallacies and biases. On the other hand, a *contaminated mindware* can verify when adolescents adhere to superstitious thinking, i.e., the propensity of having beliefs based on perceiving biased casual relationships between unrelated events [30], especially to superstitious beliefs about winning, i.e. the beliefs that one can control random events [31]. Thus, a *contaminated mindware* is read as more due to the presence of *affective risk factors*, as superstitious beliefs are linked to affective conditioning invalidating human rationality being a thinking disposition that can affect reasoning regardless of cognitive abilities [32]. Taken together, low levels of *cognitive protective factors* and high levels of *affective risk factors* can explain for the mechanisms under which gambling-related cognitive distortions arise in adolescents and lead to high levels of gambling frequency and problem gambling.

Based on the empirical evaluation of the above described model, Donati et al. [11] developed and tested the efficacy of educational activities aimed at enhancing cognitive protective factors, in particular probabilistic reasoning ability, and at reducing affective risk factors, i.e., superstitious thinking. Two training units were developed, each one consisting in a series of activities having a specific procedure: Introduction, where conductor gave the instructions, individual work done by each student, collective discussions among participants guided by the expert, and finally a closure with a reworking/summary of the contains emerged during the activity. This way of proceeding allowed to bring out the preconception of students, to highlight the eventual wrong conception, to explain and to reinforce the right one. This strategy followed the *conceptual change model* which explains that a prerequisite for new conception can be acquired if the old one is perceived as unsatisfactory [33]. The educational activities were evaluated using an experimental design. Findings showed that the activities were able to reduce gambling-related cognitive distortions. Moreover, a reduction of gambling frequency over a six-months period was obtained.

Following these premises, the aim of this work was to employ the above described model inside a large-scale dissemination preventive program involving a large amount of youth [34].

To ensure the effectiveness of the intervention in the school context, we conducted a first study (Study 1) to develop and monitor a training course for the intervention providers. Psychologists and health educators were defined as the intervention providers to work in the school and that had to be trained about the conceptual and practical model, consistent with the suggestions that gambling specialist should be preferred to school teachers [8, 9]. Following the specific guidelines [21], the training process was thought to be characterized by a training phase and a coaching phase, with the training phase which was assessed in its efficacy. The goal of the training course was enhancing psychologists and educators' knowledge and practical competence about the psychological dimensions addressed through the intervention. In detail, concerning the cognitive protective factors, the training course was directed on fostering intervention providers' knowledge about gambling meaning and functioning, and to make them able to implement in the classrooms of the educational activities aimed at modifying cognitive factors (First Didactic Unit). Regarding the affective factors, the training course was aimed at reducing intervention providers' susceptibility to probabilistic reasoning biases, and to transmit them the necessary competences to conduct with the students the activities aimed at modifying affective risk factors (Second Didactic Unit). As for the organizational aspects, intervention providers were educated about the methodological and procedural steps to be implemented in the program. Concerning the didactic techniques to be applied in the classrooms, the course was finalized to teach them the fundamentals of the conceptual change model [33]. We also aimed at verifying that the interventions providers were satisfied for the training course received, and they felt high levels of self-efficacy in implementing the educational activities with adolescents.

After having verified the adequacy of the training course for the intervention providers, with a second study (Study 2), we aimed at monitoring the educational activities implemented in PRIZE through two didactic units. The goal was to enhance some protective cognitive factors, i.e., correct gambling knowledge, randomness understanding, and probabilistic reasoning, and to reduce some affective risk factors, i.e., superstitious thinking and economic positive gambling outcome expectation, among adolescents. We also aimed at verifying a reduction of gambling-related cognitive distortions in the short-term, both as self-reported thoughts, and as behavioural erroneous decisions in fictitious gambling contexts. The goal was to promote a reduction of gambling behaviour in the long-term. To monitor these predicted results, three measurement sessions were planned: A pre-test, one week before the beginning of the intervention, and a post-test at about one week after the conclusion of the intervention, and a 4-5 months follow-up.

## Study 1

The adequacy of the training course for the intervention providers was tested in different ways. First, to assess the change of the cognitive and affective factors, a comparison between a pre-test and a post-test session was conducted. We hypothesized that participants, after a series of training meetings, would have increased correct knowledge about gambling and reduced susceptibility to probabilistic reasoning biases. We also evaluated the participants' degree of learning of competencies necessary to conduct in classes the educational activities included in the program, both those related to cognitive and affective factors. Moreover, the participants' satisfaction for the training received and self-efficacy were assessed.

## Materials and methods

### Participants

The intervention providers were 44 health professionals (32 females, $M_{age}$ = 39.34 years, $SD$ = 7.38, range = 24–58). The jobs carried by the health professionals were heterogeneous: 28

were Professional educators or Psychologists, 11 were Psychotherapists, and 5 were other health professional figures. Regarding the educational qualification, the majority of participants ($n$ = 33) had a university degree, while three possessed the high school diploma, and eight had gained a post-university qualification. The majority (68%) had previous experiences in school contexts (e.g., conduction of projects or in role of substitute teacher). In addition, more than half of them (56%) had past experience in the gambling field, especially in clinical treatment of pathological gamblers.

## Design and procedure

The training course was implemented from September 2019 to December 2019, and it was organized into seven meetings, four of which were of approximately four hours each, while three encounters have committed about eight hour each. All the meetings were carried out using face-to-face mode. During the training course, heterogeneous didactic methodologies/techniques were used: Frontal lessons, group discussions or exercises, training role-playing/simulations. These meetings were carried out with the aid of slides in .ppt, which were subsequently delivered in a paper form to each participant to allow an individual study. During the frontal lessons, the conceptual change model was used as teaching strategy. Firstly, the trainees' preconceptions of the theme dealt were analysed; then, any wrong conception was highlighted and modified through the explanation and the reinforcement of the right one. Furthermore, in the frontal lessons, ample space was left for any questions or curiosities from the trainees during and at the end of each explanation. Group discussions and exercises were useful moments to allow participants to become familiar with the materials/instruments, techniques, procedures, contents of the program, and to encourage the interaction among health professionals. These goals were pursued using, in some cases, technique as simulations or role-playing. In particular, in the first step of the training course, the trainers impersonated the role of educators in classroom during different situations (e.g., perform a part of intervention in class), while the participants simulated the role of students. Subsequently, through the use of role-playing, participants took on the role of educators themselves and directly implemented various activities foreseen in the program (e.g., talk with teacher/schools' principal to define the schedule of interventions at school). The use of role-playing allows to switch from the theoretical to the experiential and practical side [35]. In the training role-playing, certain participants take on a role and simulate operationally specific events while others observe what happens; this instrument allows an experiential learning considering at the same time the affective and relation dimensions. However, during the entire training course, the active participation of trainees was encouraged as much as possible.

To evaluate the training course, different measurements sessions were organized as a function of the dimensions to be evaluated, by previously obtaining informed consent. Ethical approval for administering self-report instruments to the intervention providers in order to evaluate the efficacy of the training course was a prerequisite of the project (Resolution of the Tuscany Region n. 771, 9 July 2018), thus it was not necessary to seek for a new ethical approval. In order to verify the change of cognitive protective factors and affective risk factors, a pre- and post-test session was organized, i.e., respectively at the second and fifth meeting of the training course. Additionally, to evaluate participants' competency in conducted the educational activities on cognitive and affective factors in the classrooms, an *ad-hoc* achievement test was developed for the post-test session. Moreover, at the end of the training course, participants' satisfaction for the training contents and self-efficacy were assessed. All data were collected anonymously.

Participants were trained to be able to conduct the PRIZE program in five meetings for each school class, i.e., two corresponded to the intervention declined in two didactic units,

while three included program evaluation sessions organized in a pre-, post-test and in a follow-up. Moreover, participants were instructed on temporal (i.e., distance between the two encounters) and spatial (i.e., in classrooms) organizational aspects of the two units, on the procedural steps followed to perform the various activities, and on the measurement scales used with students. Furthermore, the participants were provided to a "written script" for each meeting to be implemented, that served as a guide for them. Finally, to favour the dissemination, in PRIZE, the educational activities was planned to be conducted by a couple of providers for each classroom: In each meeting, alternately, one of them had to directly drive the activities, while the other one observed the interactions of the students and in general helped/assisted the conduction (i.e. wrote any notes in board-diary). The specific objectives for each meeting are proposed in Table 1.

To collect socio-demographic information about participants, gender, age, occupation, educational qualification, and previous job experience in the field of gambling were collected.

**Pre- and post-test instruments.** To assess participants' correct knowledge of gambling, the *Gambling Related Knowledge Scale – For Adolescents* (GRKS – A) [36] was used. This scale consists of eight items Likert-type items using a four-point scale that ranging from 1= strongly disagree to 4 = strongly agree. An example item is: "*Gambling can be a dangerous activity*". The GRKS – A total score was obtained from the sum of the scores of each item that composes the scale. Higher scores correspond to high correct knowledge about gambling features.

To assess participants' susceptibility to biases in probabilistic reasoning, a series of tasks were administered following existing scales [e.g., 37]. Each task had three possible answers

**Table 1. Objectives for each meeting of the training course for the intervention providers.**

| Meeting | Objectives |
|---|---|
| 1st | • Introducing the training course's participants;<br>• Introducing and opening of the training course;<br>• Increasing knowledge about gambling prevention in the school context;<br>• Transmitting knowledge of the explanation and intervention reference model. |
| 2st | • Increasing knowledge of the organization of the program;<br>• Obtaining informed consents by the participants and carrying out an initial assessment (i.e., pre-test);<br>• Incrementing knowledge about the reference model;<br>• Explaining pre-test assessment measures for the adolescents in the intervention implementation. |
| 3st | • Emphasizing the importance of having high levels of protective cognitive factors in doing gambling prevention programs with youth;<br>• Improving participants' correct knowledge about gambling;<br>• Transmitting knowledge about the educational activities included in the First Didactic Unit in PRIZE;<br>• Developing practical skills to manage the First PRIZE Didactic Unit. |
| 4st | • Emphasizing the importance of minimizing affective risk factors in doing gambling prevention programs with youth;<br>• Reducing participants' susceptibility to probabilistic reasoning biases;<br>• Transmitting knowledge about the educational activities included in the Second Didactic Unit in PRIZE;<br>• Developing practical skills to manage the Second PRIZE Didactic Unit. |
| 5st | • Developing knowledge and skills to manage the early stages of PRIZE program;<br>• Informing participants about the couples of intervention providers and assigned schools;<br>• Carrying out a final assessment (i.e., post-test);<br>• Deepening the theoretical knowledge of the intervention providers providing bibliographical references. |
| 6st | • Disseminating the results about the evaluation of the training course;<br>• Monitoring the ongoing achievement of the participants about knowledge and competences;<br>• Giving feedback to resolve any participants' problems or doubts. |
| 7st | • Monitoring the intervention providers' ongoing implementation of the program;<br>• Defining/Organizing the subsequent steps;<br>• Giving feedback to resolve any participants' problems or doubts;<br>• Assessment of participants' satisfaction and self-efficacy toward the training course. |

https://doi.org/10.1371/journal.pone.0266825.t001

each, of which only one results to be correct. A total score, summing the erroneous responses, was computed. An example of item is: "*A marble bag contains 15 blue and 15 green marbles. After you drew 5 marbles (the marble drawn was always put back into the bag), a sequence of 5 green marbles was obtained. What is the most likely outcome if a marble is drawn a sixth time*? *a. a green marble*, *b. a blue marble*, *c. blue and green are equally likely*." In this task, the a. option response corresponds to the hot hand bias [38], the b. option response to the gambler's fallacy [39], while the c. option response was the correct one.

**Evaluation of achievement.** To verify participants' learning of the competencies necessary to implement the educational activities in the classrooms, an *ad hoc* achievement test was developed. Four items were related to the educational activities focused on cognitive dimensions, and other four items regarded the educational activities focused on affective dimensions. Each item included three possible answers, among which only one was correct (each response was scored as equal to 1 if correct or 0 if wrong, so that the total score ranged from 0 to 4). An example of item evaluating achievement on activities more cognitive oriented was: "*What are the characteristics that gambling activities have in common*?. *a. The fact that these are case-based activities. b. The fact that there is a prize up for grabs. c. The fact that these are activities based on chance*, *a bet on material goods is made and there is a prize up for grabs*". An example of item evaluating achievement on activities more affective oriented was: "*In "hot" contexts*: *a. Only those who do not have adequate knowledge tend not to apply the rules of reasoning. b. Even if you know the rules*, *it is possible not to apply them due to the emotional salience of the situation. c. It is very likely to adhere to the rules of reasoning in making decisions*".

**Assessment of satisfaction toward the training course and self-efficacy.** An evaluation sheet focused on the training course was administered. This questionnaire consisted of six items with a five-point Likert scale (i.e., "*How satisfied are you with the contents covered in the course*?" with a response scale ranging from 1= *Very dissatisfied* to 5= *Very satisfied*). In detail, the questionnaire explored the personal satisfaction degree regarding four areas (i.e., contents treated, didactic modalities and materials, organizational aspects of the meetings), the perception of the utility of the contents in providing skills about gambling prevention in adolescents, and perceived self-efficacy. Moreover, two open-ended questions on the less and more interesting aspects of the course were included. Participants could also report any observations and suggestions. In order to stimulate sincere responses as much as possible, we asked participants to do not report their code on the sheet in order to make it anonymous.

## Results

Thirty-eight participants (89%) ended the training course. There were no differences between the participants that completed the training course and those who did not complete the training course concerning gender ($p$ = .129, $\varphi$ = .720) and age ($p$ = .074, Cohen's $d$ = .65). To verify whether the training course had an effect on correct gambling knowledge and susceptibility to probability biases, a $t$-test for paired samples was performed (i.e., comparing pre-test and post-test scores for each variable) adjusting the level of significance by Bonferroni correction to .025 (.05/2). A significant difference from pre- to post-test, associated with a medium effect size – was obtained for correct gambling knowledge, which resulted to improve. Susceptibility to probabilistic biases significantly change over time too. In particular, a large effect size was for vulnerability to probability biases from pre-test to post-test (Table 2).

To assess participants' achievement, we analysed their responses at the *ad-hoc* test. Mean scores obtained to cognitive- and affective-oriented items, as well as the percentage of correct answers for each item, were calculated. Results showed a high average score for both the item groups (Cognitive-oriented items: $M$ = 3.78, $SD$ = .63; Affective-oriented items: $M$ = 3.75,

**Table 2. Mean scores compared with paired-samples *t*-test (and related effect sizes) at pre- and post-test.**

| | Pre-test | | Post-test | | $t(df)$ | $p$ | Cohen's $d$ |
|---|---|---|---|---|---|---|---|
| | *M* | *SD* | *M* | *SD* | | | |
| Correct gambling knowledge | 28.83 | 3.56 | 30.43 | 2.19 | - 2.76(22) | .011 | .58 |
| Susceptibility to probability biases | 2.71 | 2.36 | .69 | .99 | 5.38(34) | <.001 | .91 |

https://doi.org/10.1371/journal.pone.0266825.t002

*SD* = .87), indicating that the sample actually acquired the addressed competences. The percentage of correct answers for each item ranged from 83% (i.e., identification of an impulsive behaviour) to 97% (i.e., independence of multiple random events), indicating that all the questions had obtained a majority of correct answers (Table 3).

Concerning the participants' evaluation of the training meetings related to the main contents of PRIZE program, results showed that the mean scores observed (*M* = 23.60, *SD* = 4.19) was higher that the theoretical one (i.e., 18). Moreover, high scores were observed in all the domains considered: contents treated, didactic modalities and materials, organizational aspects of the meetings, usefulness of the contents in providing skills about the gambling prevention in adolescents. and self-efficacy about the possibility of doing the actions required by the programs (Table 4).

As regards the most interesting aspects of the meetings, participants reported several factors such as some theoretical (e.g., on the probabilistic reasoning and on cognitive distortions) and practical elements (e.g., use of simulations or interactive lessons), the organization of the course (e.g., division of the course into a practical and theoretical part), and the opportunity to learn a structured way of doing gambling prevention at school. As the less interesting aspects of the meetings, they indicated the redundancy of some contents (e.g., topics or simulations) and the focus on certain theoretical aspects (e.g., gambling prevention).

## Discussion

The aim of this study was to evaluate the training course with the intervention providers inside the PRIZE program. To that aim, we verified changes from the pre-test to the post-test session. As hypothesized, we found a significant increase of correct knowledge about gambling and a significant reduction of the susceptibility to probability biases. These changes were respectively medium and high in size, attesting that a practical acquisition really happened. We also checked that participants actually acquired the main competences to implement in the school classes both the cognitive-oriented and the affective-oriented activities. In this regard, although we verified that more than 80% of the participants responded correctly to all the questions about the cognitive and the affective areas, for the cognitive area, all the questions reached

**Table 3. Percentage of correct responses for each item of the achievement test.**

| *Item's content* | *Percentage of correct responses (%)* |
|---|---|
| *Cognitive area* | |
| Gambling features | 95 |
| Independence of multiple random events | 97 |
| Evaluation of sample space | 95 |
| Probability of winning (%) in a scratch cards | 92 |
| *Affective area* | |
| Reasoning in "hot" decision-making context | 87 |
| Gambling-related cognitive distortions | 89 |
| Concept of losses in the gamble of scratch cards | 89 |
| Identification of an impulsive behaviour | 83 |

https://doi.org/10.1371/journal.pone.0266825.t003

**Table 4. Mean scores for each item of the training course's satisfaction test.**

| | *M* | *SD* |
|---|---|---|
| Contents of the meetings | 4.17 | .79 |
| Didactic modalities | 4.03 | .89 |
| Didactic materials | 3.97 | .85 |
| Organizational aspects of the meetings | 3.57 | 1.07 |
| Usefulness of the contents in providing skills | 4.03 | .76 |
| Self-efficacy | 3.83 | .65 |

https://doi.org/10.1371/journal.pone.0266825.t004

more than 90% of correct responses, while for the affective area, percentages of correct responses did not reach 90%. This fact may be due by the accentuated human susceptibility to cognitive biases, cognitive distortions and impulsive responses, even for adult experienced people [38]. Finally, participants' satisfaction for the all the aspects relative to the training course received was high, in particular their self-efficacy in implementing the educational activities with the adolescents. Overall, findings suggested that the operators were able to be the PRIZE providers in the schools as they showed to have acquired the necessary knowledge, competence, skills, and self-efficacy to conduct the prevention program.

Although assessing the effects of training courses for the people who implement the educational interventions with students represents a novelty in the adolescent gambling research field, the obtained findings are in line with those evidenced in some studies testing the efficacy of trainings addressed to employees who work in the gambling context [39, 40], in which learning of the training contents was observed. Moreover, the above mentioned result concerning participants' satisfaction towards the training course is important as the training satisfaction seems to influence work commitment [41] and job satisfaction [42]. Performance in work appears also associated with self-efficacy [43], which resulted to be high in this study, too. Indeed, participants declared to feel able to manage the different phases/aspects of the program at the end of the training course. Thus, to summarize, this study delivers for the first time data on the adequacy of a training course capable of preparing health professionals to provide a gambling prevention program based on a theoretical model.

## Study 2

As a second step, we evaluated the effectiveness of the intervention conducted in the classrooms by the intervention providers who have attended the training course. In particular, in the short-term, we hypothesized that students would enhance protective cognitive factors, i.e., correct gambling knowledge, randomness understanding, and probabilistic reasoning ability, and that they would reduce affective risk factors, i.e., superstitious thinking and economic positive gambling outcome expectation. We also predicted to obtain a reduction of gambling-related cognitive distortions in the short-term, and a reduction of gambling behaviour in the long-term. Preliminarily, we were interested in analyzing gambling behaviour and problem gambling severity, before the implementation of the intervention.

## Materials and methods

### Participants

The institutional review boards for each school – composed by the school principal, the teachers, and the students' parents – approved the study. Then the students received an information sheet, which assured them that the data obtained would be handled confidentially and anonymously, and they were asked to give written informed assent. Parents of minors were required

to provide consent in addition to the child agreeing to participate. Parents were informed that the survey was anonymous, and confidentiality of information was assured, according to the provisions of General Data Protection Regulation (GDPR 679/2016).

A total of 1894 high school students (61% males) with a mean age of 15.68 years (*SD* = .71, range: 14–19) participated in the pre-test. Of this sample, 24% of adolescents (*n* = 428) had a migration background, especially originating from Eastern Europe, Asia, and Latin America. Participants attended 34 different institutes located in all the Tuscany Region (Italy); specifically, 96% of adolescents attended high school (40% a technical school, 39% a lyceum, and 17% a professional institute), and 4% professional training center.

Among those students, 900 (56% males, mean age = 14.58 years, *SD* = .67) were eligible to verify the short-term effects of the PRIZE program, i.e., they completed the pre-test, the first and second didactic units, and the post-test. The evaluation of the long-terms efficacy was conducted considering the students who attended also the follow-up, i.e., 662 adolescents (58% males, mean age = 15.57 years, *SD* = .60).

## Design

To verify those effects, three measurement sessions were planned: A pre-test conducted one-two months before the intervention, which occurred with two didactic units carried out within 15 days of each other, a post-test within 15 days after the end of the intervention activities, and a follow-up within 3-4 months by the intervention. At the pre-test, gambling behavior and related problem gambling symptoms, protective cognitive factors, risk affective factors, and gambling-related cognitive distortions were assessed. At the post-test, the above cited dimensions were measured with the exception of gambling habits and problematic behavior, that were assessed at the follow-up.

## Measures and procedure

**Cognitive protective factors.** Gambling-related correct knowledge was measured through the *Gambling Related Knowledge Scale – For Adolescents* (GRKS-A) [36], the self-report scale used in Study 1. It was developed for Italian adolescents and it has good psychometric properties. Coefficient alpha for the current sample at the pre-test was sufficient (α = .70) and very good at the post-test (α = .88).

Random events knowledge was assessed by using the Italian version of the *Random Events Knowledge Test – Youth Version* (REKT – YV) [44, 45]. The scale is composed of twenty-two items with a 4-point Likert-type response scale ranging from 1 (totally disagree) to 4 (totally agree). A sample item is "*A random looking number (e.g., 12 – 5 – 23 – 7 – 19 – 34) is more likely to win than a number that has a sequence in it (e.g., 1- 2- 3- 4 – 5 – 6)*". High scores at the REKT – YV correspond to greater knowledge of randomness. The coefficient alpha for the current sample was satisfactory at the pre-test (α = .70) and very good at the post-test (α = .81).

Probabilistic reasoning ability was detected with the *Non-Gambling Task* (NGT) [46]. In this task, a table with six different sequences of six coin tosses is presented (with two possible events: T = Tail, H = Head). For each sequence, the respondents were asked to indicate the probability of T if a further seventh roll is made. For each correct answer, one point is given. High scores indicate good probabilistic reasoning skills. The coefficient KR-20 was excellent both at the pre-test (α = .92) and post-test (α = .96).

## Affective risk factors

To measure superstitious thinking, the Italian version [47] of the *Superstitious Thinking Scale* (STS) [48] was used. It is composed of eight Likert-type items using a 5-point scale ranging from totally false to totally true. Higher scores represent high levels of superstitious thinking.

An example of an item is "*The number 13 is unlucky*". Coefficient alpha for the current sample was good at the pre-test (α = .82) and post-test (α = .87).

Positive expectations towards the economic advantages related to gambling was assessed through the *Money* subscale of the *Gambling Expectancies Questionnaire* (GEQ) [49], revised in a modified form and adapted for Italian adolescents (GEQ – MOD) [6]. In comparison with the original scale, the GEQ – MOD can be administered to all adolescents, regardless of their gambling behavior, and it is composed of Likert-type items with a 5-point scale (rather than a seven-point Likert scale). An example of item of the *Money* subscale is "*If you were gambling, gambling would make you. . . Getting rich*". Coefficient alpha for the current sample was very good at the pre-test (α = .88) and the post-test (α = .81).

## Gambling-related cognitive distortions

Cognitive distortions related to gambling were assessed in two different ways. A self-report scale about erroneous thoughts about gambling outcomes was administered together with a behavioral choice task.

The *Gambling Related Cognitions Scale – Revised for Adolescents* (GRCS – RA) [50] is a self-report scale to assess gambling-related cognitions in young people, adapted from the original GRCS [26] for Italian adolescents [51]. In comparison with the original instrument, the revised form for adolescents contains fourteen Likert-type items having a 5-point scale ranging from 1 (strongly disagree) to 5 (strongly agree). Three specific gambling-related biases, according to Toneatto's model [52], are measured by the following subscales: *Illusion of Control* (4 items), *Predictive Control* (6 items), and *Interpretative Bias* (4 items). In order to make the scale more appropriate and suitable for adolescents, items have been modified as to obtain third person formulations (rather than first-person) that therefore could be administered to young people regardless their gambling behavior, contrary to the original scale, which was developed to be administered with adult gamblers. Examples of items are "*Specific numbers and colors can help increase the chances of winning in gambling*" (*Illusion of Control*), "*In gambling, if you win once, you will surely win again*" (*Predictive Control*), and "*In gambling, people continue gambling despite losses because losses are due to bad luck and bad circumstances*" (*Interpretative Bias*). The GRCS - RA total score, obtained by summing the score for each item, was calculated. The coefficient alpha was good at the pre-test (α = .82) and excellent at the post-test (α = .93).

A behavioral choice task was also used to assess cognitive distortions related to gambling intended as fallacious decisional choices in a fictitious gambling context. In detail, the *Gambling Task* (GT) [46] was administered. In this task, participants were presented with the same outcome sequences as the NGT, but, for each sequence, they were asked to indicate how much money, from a minimum of €0 to a maximum of €10 (available for each sequence), would bet on Tails if a seventh toss would be made. A net score was calculated by subtracting the average amount of money bet on the first, the third, and the fourth sequence (i.e. those sequences which make more likely to bet on Tails according to the gambler's fallacy bias) from the average amount of money bet on the second, the fifth, and the sixth sequence (i.e. those sequences which make less likely to bet on Tail according to heuristic strategies). Thus, higher scores corresponded to high levels of susceptibility to the gambler's fallacy. Fallacious choices are operationalized as those that implied an amount of betting depending on the previous sequence of outcomes, as in the case of the gambler's fallacy, consisting in betting a higher amount of money on those sequences ending with Heads, or the hot hand bias, i.e., betting a higher amount of money on Tail as this event was judged as more likely than Head based on the relative frequency of occurring in the sequence. The coefficient KR-20 was very good at the pre-test (α = .89) and post-test (α = .88).

## Gambling behavior

To investigate gambling behavior, we administered the *Gambling Behaviour Scale for Adolescents* (GBS-A) [53]. The GBS-A is composed of two sections. The first section consists of unscored items investigating gambling frequency. Ten items assess the frequency (never, sometimes in the year, sometimes in the month, sometimes in the week, daily) of participation during the last year in ten gambling activities (card games, bets on games of personal skill, bets on sports games, bets on horse races, bingo, slot machines, scratch cards, lotteries, online games and private bets with friends). Based on their responses to this section, participants were identified as *non-gamblers* (no gambling behaviour) or *gamblers* (gambling on at least one activity). The second section consists of nine scored items assessing the *Diagnostic and Statistical Manual of Mental Disorders* (DSM-5) [54] diagnostic criteria for Gambling Disorder (GD). Each item is evaluated on a three-point Likert scale ranging from 0 (*Never*) to 2 (*Often*). Based on the responses to this section, it is possible to derive an Item Response Theory-based score for each respondent. Following this IRT-based scoring procedure, respondents can be classified into *non-problem gamblers*, *at-risk gamblers* and *disordered gamblers*. The GBS-A has been shown to be unidimensional and useful for mid- to high levels of GD severity [55]. In this study, Cronbach's alpha was satisfactory ($\alpha = .74$).

The scales were administered by the trained operators and individually during class time, in this order: NGT, STS, GRKS-A, REKT – YV, GT, GRCS-RA, GEQ-MOD – Money, and GBS-A at the pre-test. Students were provided with a brief introduction to the project and with instructions on completing the surveys and were assured confidentiality. Answers were collected in a paper-and-pencil format and data collection was completed in approximately 40-50 min at the pre-test, 30-35 min at the post-test, and 15-20 min at the follow-up.

## Results

**Gambling behavior and problem gambling at the pre-test.** The results indicated that 75% of the respondents ($n = 1421$) have gambled at least once in the past 12 months. The activities that were most frequently engaged in were scratch cards (48%), bingo (47%) and private bets with friends (37%). The majority of adolescent gamblers (60%) gambled on land-based activities. On average, gamblers practiced about two gambling activities ($M = 2.5$, $SD = 2.2$).

Concerning problem gambling, 82% were non-problem gamblers, 12% were identified as at-risk gamblers and 6% were problem gamblers. For analyses purposes, at-risk and disordered gamblers were collapsed in the same group (At-risk and Problem Gamblers; ARPGs), following some previous studies [55], and it was compared with the group of Non-At-risk and Problem Gamblers (NARPGs). We found a significant difference in the distribution of the two categories of gamblers across gender ($\chi^2(1) = 21.65$, $p < .001$, $\varphi = .13$) and immigration status ($\chi^2(1) = 17.83$, $p < .001$, $\varphi = .12$). Indeed, among male adolescents, there was a higher prevalence of ARPGs (21%), with respect to female adolescents (ARPGs = 12%). A greater problematic status was also observed considering the immigrant adolescents (ARPGs = 26%), with respect to the non-immigrant adolescents (ARPGs = 16%).

**Intervention's short-term effects.** Preliminarily, we verified if there were significant differences in terms of gender and age between adolescents that participated in the pre-test, completed the intervention didactic units and the post-test, and the participants that drop out from the intervention study. Significant but very small in size differences emerged for gender ($\chi^2(1) = 15.15$, $p<.001$, $\varphi = .09$), with 56% of males among those who completed the pre-test, the intervention, and the post-test, and 65% of males among the participants who did not complete the short-term evaluation. Even for age, the detected difference among those who completed the pre-test, the intervention, and the post-test ($M = 15.62$, $SD = .67$) and the

participants who did not complete the short-term evaluation (M = 15.72, SD = .75) was significant but very small in size (t(1842) = 2.80, p = .003, Cohen's d = .13).

Short-term intervention effects were tested performing paired *t*-test across pre- and post-test for cognitive protective factors, affective risk factors, and gambling-related erroneous thoughts, and by comparing the prevalence of fallacious choices at the GT from the pre-test to the post-test through $\chi^2$ tests.

As expected, results showed a significant increase of adolescents' cognitive protective factors, and a significant reduction of affective risk factors, adjusting the level of significance by Bonferroni correction to .008 (.05/6). Moreover, gambling-related erroneous thoughts were significantly reduced. To note that the change over time was characterized by a large effect size for random events knowledge and probabilistic reasoning ability, and by a medium effect size for superstitious thinking, monetary positive outcome expectation, and gambling-related erroneous thoughts (Table 5).

Moreover, a significant change ($\chi^2(1) = 17.00$, $p$ <.001, $\varphi = .15$) of the prevalence of fallacious choices at the GT occurred from the pre-test (normative choices = 33% and fallacious choices = 67%) to the post-test (normative choices = 84% and fallacious choices = 16%), indicating a reduction of erroneous choices.

To check if the detected changes found in the total sample occurred in male as well as female adolescents and in younger as well as older participants, we conducted the above reported analyses separately for gender (males, females) (Table 6) and age (splitting the sample along the median value of 15.50 years) groups (Table 7).

Moreover, a significant change of the prevalence of fallacious choices at the GT occurred from the pre-test to the post-test occurred both among boys ($\chi^2(1) = 11.75$, $p < .001$, $\varphi = .16$; Pre-test: normative choices = 37% and fallacious choices = 63%, Post-test: normative choices = 83% and fallacious choices = 17%), and among girls ($\chi^2(1) = 8.71$, $p = .003$, $\varphi = .16$; Pre-test: normative choices = 26% and fallacious choices = 74%, Post-test: normative choices = 85% and fallacious choices = 15%), indicating a reduction of erroneous choices in male as well as female adolescents. Concerning age, a significant change of the prevalence of fallacious choices at the GT occurred from the pre-test to the post-test occurred both among younger adolescents ($\chi^2(1) = 8.80$, $p = .003$, $\varphi = .14$; Pre-test: normative choices = 33% and fallacious choices = 67%, Post-test: normative choices = 84% and fallacious choices = 16%), and among older adolescents ($\chi^2(1) = 8.90$, $p = .003$, $\varphi = .17$; Pre-test: normative choices = 30% and fallacious choices = 70%, Post-test: normative choices = 83% and fallacious choices = 17%), indicating in both the groups a reduction of erroneous choices.

**Gambling behavior and problem gambling at the follow-up.** First, we looked at the descriptive statistics concerning gambling behavior at the follow-up session. The results

**Table 5. Mean scores compared with paired-samples *t*-test (and related effect sizes) at pre- and post-test ($n = 900$).**

| | Pre-test | | Post-test | | *t*(*df*) | *p* | Cohen's *d* |
|---|---|---|---|---|---|---|---|
| | *M* | *SD* | *M* | *SD* | | | |
| *Cognitive protective factors* | | | | | | | |
| Correct gambling knowledge | 27.35 | 3.73 | 28.35 | 2.19 | - 7.17 (890) | <.001 | .24 |
| Random events knowledge | 60.59 | 6.47 | 67.90 | 8.80 | -27.75 (887) | <.001 | .93 |
| Probabilistic reasoning ability | 3.38 | 2.42 | 5.67 | 1.25 | -26.35 (844) | <.001 | .91 |
| *Affective risk factors* | | | | | | | |
| Superstitious thinking | 18.51 | 6.74 | 16.99 | 7.21 | 8.63 (885) | <.001 | .29 |
| Monetary positive outcome expectation | 9.23 | 3.41 | 8.02 | 3.35 | 9.33 (883) | <.001 | .31 |
| Gambling-related erroneous thoughts | 27.51 | 8.94 | 24.01 | 10.15 | 10.04 (886) | <.001 | .27 |

https://doi.org/10.1371/journal.pone.0266825.t005

**Table 6. Mean scores compared with paired-samples *t*-test (and related effect sizes) at pre- and post-test by gender (*n* = 900).**

| *Cognitive protective factors* | Correct gambling knowledge | | | | | | |
|---|---|---|---|---|---|---|---|
| | Pre-test | | Post-test | | *t*(*df*) | *p* | Cohen's *d* |
| | *M* | *SD* | *M* | *SD* | | | |
| Males (n = 503) | 26.92 | 3.94 | 27.75 | 4.50 | - 420 (502) | <.001 | .19 |
| Females (n = 397) | 27.87 | 3.42 | 28.97 | 3.43 | - 5.78 (396) | <.001 | .29 |
| | Random events knowledge | | | | | | |
| | Pre-test | | Post-test | | *t*(*df*) | *p* | Cohen's *d* |
| | *M* | *SD* | *M* | *SD* | | | |
| Males (n = 501) | 61.38 | 6.82 | 68.06 | 9.23 | -18.11 (502) | <.001 | .81 |
| Females (n = 396) | 59.47 | 5.82 | 67.48 | 8.31 | - 22.10 (395) | <.001 | 1.11 |
| | Probabilistic reasoning ability | | | | | | |
| | Pre-test | | Post-test | | *t*(*df*) | *p* | Cohen's *d* |
| | *M* | *SD* | *M* | *SD* | | | |
| Males (n = 481) | 3.86 | 2.43 | 5.71 | 91.19 | -16.27 (480) | <.001 | .74 |
| Females (n = 375) | 2.74 | 2.24 | 5.56 | 1.40 | - 22.21 (374) | <.001 | 1.15 |
| *Affective risk factors* | Superstitious thinking | | | | | | |
| | Pre-test | | Post-test | | *t*(*df*) | *p* | Cohen's *d* |
| | *M* | *SD* | *M* | *SD* | | | |
| Males (n = 500) | 17.18 | 6.65 | 15.54 | 6.92 | 6.89 (499) | <.001 | .31 |
| Females (n = 395) | 20.28 | 6.43 | 18.99 | 7.14 | 5.24 (394) | <.001 | .26 |
| | Monetary positive outcome expectation | | | | | | |
| | Pre-test | | Post-test | | *t*(*df*) | *p* | Cohen's *d* |
| | *M* | *SD* | *M* | *SD* | | | |
| Males (n = 497) | 8.96 | 3.50 | 8.19 | 3.46 | 4.46 (496) | <.001 | .20 |
| Females (n = 396) | 9.53 | 3.31 | 7.89 | 3.23 | 8.61 (395) | <.001 | .43 |
| | Gambling-related erroneous thoughts | | | | | | |
| | Pre-test | | Post-test | | *t*(*df*) | *p* | Cohen's *d* |
| | *M* | *SD* | *M* | *SD* | | | |
| Males (n = 501) | 26.73 | 8.96 | 24.39 | 10.19 | 5.17 (500) | <.001 | .23 |
| Females (n = 395) | 28.59 | 8.83 | 23.81 | 10.17 | 9.10 (394) | <.001 | .35 |

https://doi.org/10.1371/journal.pone.0266825.t006

indicated that 60% of the respondents (n = 398) have gambled at least once in the last 2 months. The activities that were most frequently engaged in were online gambling games (25%), scratch cards (18%), and bingo (15%). On average, gamblers practiced about one gambling activity (M = 1.4, SD = 1.7).

Concerning problem gambling, 89% were NARPGs and 11% were ARPGs. A significant difference in the distribution of the two categories of gamblers across gender ($\chi^2(1) = 7.65$, $p = .006$, $\varphi = .11$) was found. Indeed, among male adolescents, there was a higher prevalence of ARPGs (13%), with respect to female adolescents (ARPGs = 6%). Non-significant differences were found across immigration status ($\chi^2(1) = .06$, $p = .815$, $\varphi = .01$).

**Intervention's long-term effects.** Prior to conduct the long-term evaluation analyses, we verified if there were significant differences in terms of gender and age between adolescents that participated in the pre-test, completed the intervention didactic units, the post-test, and the follow-up sessions, and the participants that drop out from the intervention study for the long-term evaluation. A non-significant difference was found for gender ($\chi^2(1) = 2.38$, p=.123, $\varphi = .04$), with 58% of males among those who completed the pre-test, the intervention, the post-test, and the follow-up, and 62% of males among the participants who did not complete

**Table 7. Mean scores compared with paired-samples *t*-test (and related effect sizes) at pre- and post-test by age groups (*n* = 900).**

| *Cognitive protective factors* | Correct gambling knowledge | | | | | | |
|---|---|---|---|---|---|---|---|
| | Pre-test | | Post-test | | *t*(*df*) | *p* | Cohen's *d* |
| | *M* | *SD* | *M* | *SD* | | | |
| Younger (n = 507) | 27.41 | 3.919 | 28.85 | 3.48 | -9.31 (506) | <.001 | .41 |
| Older (n = 386) | 27.28 | 4.36 | 27.62 | 4.69 | - 1.38 (385) | .084 | .07 |
| | Random events knowledge | | | | | | |
| | Pre-test | | Post-test | | *t*(*df*) | *p* | Cohen's *d* |
| | *M* | *SD* | *M* | *SD* | | | |
| Younger (n = 507) | 61.02 | 6.27 | 68.74 | 8.51 | -22.25 (506) | <.001 | .99 |
| Older (n = 383) | 59.90 | 6.64 | 66.54 | 9.04 | - 16.45 (382) | <.001 | .84 |
| | Probabilistic reasoning ability | | | | | | |
| | Pre-test | | Post-test | | *t*(*df*) | *p* | Cohen's *d* |
| | *M* | *SD* | *M* | *SD* | | | |
| Younger (n = 486) | 3.40 | 2.44 | 5.70 | 1.19 | -19.94 (485) | <.001 | .90 |
| Older (n = 3764) | 3.28 | 2.37 | 5.55 | 1.44 | - 17.30 (363) | <.001 | .91 |
| *Affective risk factors* | Superstitious thinking | | | | | | |
| | Pre-test | | Post-test | | *t*(*df*) | *p* | Cohen's *d* |
| | *M* | *SD* | *M* | *SD* | | | |
| Younger (n = 506) | 18.26 | 6.64 | 16.97 | 7.23 | 5.62 (505) | <.001 | .25 |
| Older (n = 382) | 18.85 | 6.84 | 17.06 | 7.16 | 6.63 (381) | <.001 | .34 |
| | Monetary positive outcome expectation | | | | | | |
| | Pre-test | | Post-test | | *t*(*df*) | *p* | Cohen's *d* |
| | *M* | *SD* | *M* | *SD* | | | |
| Younger (n = 505) | 9.21 | 3.37 | 7.94 | 3.29 | 7.50 (504) | <.001 | .24 |
| Older (n = 381) | 9.23 | 3.50 | 8.17 | 3.46 | 5.24 (380) | <.001 | .27 |
| | Gambling-related erroneous thoughts | | | | | | |
| | Pre-test | | Post-test | | *t*(*df*) | *p* | Cohen's *d* |
| | *M* | *SD* | *M* | *SD* | | | |
| Younger (n = 507) | 26.791 | 8.62 | 23.25 | 9.63 | 8.42 (506) | <.001 | .28 |
| Older (n = 382) | 28.31 | 9.26 | 25.29 | 10.81 | 5.31 (381) | <.001 | .27 |

https://doi.org/10.1371/journal.pone.0266825.t007

the long-term evaluation. As for age, we found a significant but small difference (t(1842) = 4.67, p < .001, Cohen's d = .23) between those who completed the pre-test, the intervention, the post-test, and the follow-up (M = 15.57, SD = .60) and the participants who did not complete the long-term evaluation (M = 15.74, SD = .76). We also checked if the participants who completed the program until the follow-up were different from those who did not complete the study in terms of the baseline gambling frequency and severity. Results showed a non-significant difference (t(1879) = 1.38, p = .084, Cohen's d = .06) concerning gambling frequency between participants who completed the program (M = 3.47, SD = 3.75) and those who did not (M = 3.77, SD = 4.43), and a significant but small in size difference t(1408) = 3.02, p = .001, Cohen's d = .17) concerning gambling problem severity between participants who completed the program (M = 1.39, SD = 2.10) and those who did not (M = 1.81, SD = 2.66).

Long-term intervention effects were tested performing paired *t*-tests across pre- and follow-up scores for gambling frequency, gambling versatility, and gambling problem severity, and by comparing through $\chi^2$ tests the prevalence of NARPGs and ARPGs from the pre-test to the follow-up.

Results showed a significant decrease of gambling frequency, gambling versatility, and gambling problem severity, adjusting the level of significance by Bonferroni correction to .017

**Table 8. Mean scores compared with paired-samples *t*-test (and related effect sizes) at pre- and follow-up (*n* = 662).**

| | Pre-test | | Post-test | | *t*(*df*) | *p* | Cohen's *d* |
|---|---|---|---|---|---|---|---|
| | *M* | *SD* | *M* | *SD* | | | |
| Gambling frequency | 3.51 | 3.81 | 2.21 | 3.00 | 9.50 (660) | <.001 | .37 |
| Gambling versatility | 2.47 | 2.11 | 1.41 | 1.73 | 13.80 (660) | <.001 | .54 |
| Gambling problem severity | 1.43 | 2.13 | 1.01 | 2.10 | 4.28 (463) | <.001 | .20 |

https://doi.org/10.1371/journal.pone.0266825.t008

(.05/3). The changes were characterized by medium effect sizes for gambling frequency and versatility (Table 8).

Moreover, a significant change ($\chi^2(1) = 49.06$, $p < .001$, $\varphi = .33$) of the prevalence of NARPGs and ARPGs occurred from the pre-test (NARPGs = 84% and ARPGs = 16%) to the follow-up (NARPGs = 89% and ARPGs = 11%), indicating a reduction of ARPGs.

To check if the detected changes occurred in male as well as female adolescents and in younger as well as older participants, we conducted the analyses separately for gender (males, females) (Table 9) and age groups (Table 10).

A significant change of the prevalence of NARPGs and ARPGs occurred both among boys ($\chi^2(1) = 39.67$, $p < .001$, $\varphi = .31$; Pre-test: NARPGs = 80% and ARPGs = 20%, Follow-up: NARPGs = 84% and ARPGs = 16%) and girls ($\chi^2(1) = 16.59$, $p < .001$, $\varphi = .25$; Pre-test: NARPGs = 89% and ARPGs = 11%, Follow-up: NARPGs = 93% and ARPGs = 7%), indicating a reduction of ARPGs in both the gender groups. A similar result was found by age group as a significant reduction of the prevalence of NARPGs and ARPGs occurred both among younger adolescents ($\chi^2(1) = 31.66$, $p < .001$, $\varphi = .29$; Pre-test: NARPGs = 86% and ARPGs = 14%, Follow-up: NARPGs = 89% and ARPGs = 11%) and older adolescents ($\chi^2(1) = 25.72$, $p < .001$, $\varphi = .30$; Pre-test: NARPGs = 80% and ARPGs = 20%, Follow-up: NARPGs = 87% and ARPGs = 13%),

## Discussion

In order to evaluate the intervention, we tested the effects of the educational activities conducted in the classrooms by the intervention providers who attended the training course. In particular, in the short-term, we hypothesized that students participating in the program

**Table 9. Mean scores compared with paired-samples *t*-test (and related effect sizes) at pre- and follow-up by gender (*n* = 662).**

| | Gambling frequency | | | | | | |
|---|---|---|---|---|---|---|---|
| | Pre-test | | Follow-up | | *t*(*df*) | *p* | Cohen's *d* |
| | *M* | *SD* | *M* | *SD* | | | |
| Males (n = 560) | 4.22 | 4.31 | 2.64 | 3.31 | 9.35 (559) | <.001 | .40 |
| Females (n = 441) | 2.41 | 2.78 | 1.51 | 2.20 | 6.94 (440) | <.001 | .33 |
| | Gambling versatility | | | | | | |
| | Pre-test | | Follow-up | | *t*(*df*) | *p* | Cohen's *d* |
| | *M* | *SD* | *M* | *SD* | | | |
| Males (n = 560) | 2.79 | 2.26 | 1.61 | 1.86 | 13.37 (559) | <.001 | .57 |
| Females (n = 441) | 1.92 | 1.84 | 1.07 | 1.46 | 9.84 (440) | <.001 | .47 |
| | Gambling problem severity | | | | | | |
| | Pre-test | | Follow-up | | *t*(*df*) | *p* | Cohen's *d* |
| | *M* | *SD* | *M* | *SD* | | | |
| Males (n = 418) | 1.81 | 2.52 | 51.38 | 2.48 | 3.32 (480) | <.001 | . 16 |
| Females (n = 267) | 1.12 | 1.75 | .59 | 1.41 | 5.47 (266) | <.001 | .33 |

https://doi.org/10.1371/journal.pone.0266825.t009

**Table 10. Mean scores compared with paired-samples *t*-test (and related effect sizes) at pre- and follow-up by age group (*n* = 662).**

| | Gambling frequency | | | | | | |
|---|---|---|---|---|---|---|---|
| | Pre-test | | Follow-up | | *t*(*df*) | *p* | Cohen's *d* |
| | *M* | *SD* | *M* | *SD* | | | |
| Younger (n = 564) | 3.24 | 3.50 | 2.11 | 2.71 | 8.12 (563) | <.001 | .34 |
| Older (n = 427) | 3.66 | 24.22 | 2.16 | 3.19 | 8.16 (426) | <.001 | .39 |
| | Gambling versatility | | | | | | |
| | Pre-test | | Follow-up | | *t*(*df*) | *p* | Cohen's *d* |
| | *M* | *SD* | *M* | *SD* | | | |
| Younger (n = 564) | 2.37 | 2.06 | 1.38 | 1.71 | 11.68 (563) | <.001 | .49 |
| Older (n = 427) | 2.46 | 2.21 | 1.34 | 1.72 | 11.75 (426) | <.001 | .57 |
| | Gambling problem severity | | | | | | |
| | Pre-test | | Follow-up | | *t*(*df*) | *p* | Cohen's *d* |
| | *M* | *SD* | *M* | *SD* | | | |
| Younger (n = 391) | 1.31 | 2.07 | .92 | 1.92 | 3.74 (390) | <.001 | .19 |
| Older (n = 288) | 1.80 | 2.45 | 1.22 | 2.38 | 3.93 (287) | <.001 | .23 |

https://doi.org/10.1371/journal.pone.0266825.t010

would enhance protective cognitive factors, i.e., correct gambling knowledge, randomness understanding, and probabilistic reasoning, and that they would reduce affective risk factors, i.e., superstitious thinking and economic positive gambling outcome expectation. We also predicted a reduction of gambling-related cognitive distortions in the short-term, and a reduction of gambling behaviour in the long-term.

First, in line with national and international data [3, 6, 56], the large majority of adolescents resulted to be past-year gamblers, and they especially gamble on Instant scratch-cards, bingo, and private bets with friends. Most of them preferred land-based gambling activities, even if online gambling is widespread [57–59]. Following previous studies [3, 6], there was a considerable part of adolescents showing at-risk gambling and problem gambling, in particular male adolescents [60, 61] and youth with a migration background [62, 63].

Regarding the intervention's effects, we have to highlight a high attrition rate from the pre-test to the post-test (52%). In this regard, it is important to underline that we selected for the analyses only adolescents who attended both the didactic units of the intervention – analytic step that any previous study, with few exceptions [64], has been done in the previous intervention studies. Concerning the long-term, a lower attrition rate (26%) was evidenced. In the short term, in line with hypotheses, we found a significant increase of adolescents' cognitive protective factors, i.e., correct gambling knowledge, random events knowledge, and probabilistic reasoning ability, as well as a significant decrease of affective risk factors, i.e., superstitious thinking, monetary positive outcome expectation, and gambling-related erroneous thoughts and fallacious behavioral choices. Moreover, the majority of those effects were characterized by large or medium effect sizes, suggesting that the changes were practical, as well as statistically significant. We found that the above results were achieved by male as well as female adolescents and by younger and older adolescents, with the exception of correct gambling knowledge, that did not change among the group of older adolescents. This result may indicate that, for older adolescents, the current educational activities should be accompanied by other additional activities more effective with this target. However, we can underline that at the pre-test the score concerning correct gambling knowledge of this group was higher than the theorical mean at the scale, indicating a pre-existing situation of sufficient correct gambling knowledge, and our analyses indicate a persistence of this situation, without iatrogenic effects.

In the long-term, we found a significant decrease of gambling frequency, gambling versatility – with a medium effect size – and a reduction also of gambling problem severity. Those findings were verified also separately by gender and age groups.

## Conclusion

To the best of our knowledge, in literature, there is a lack of studies that have conducted and evaluated gambling preventive interventions towards adolescents by reporting and assessing the efficacy of training courses realized with the professional figures who would implement the intervention itself. The present work fills this gap by realizing a dissemination study that referred to a previous empirically tested explanation and intervention model [11]. The purpose of this work was twofold: To develop and evaluate a training intended for educators and psychologists, and to assess the short and long term effects of the intervention they implemented after receiving the training course inside the PRIZE program, in Italy.

Findings showed that the educators and psychologists who attended the training course modify themselves cognitive protective factors and risk affective factors, and acquired the necessary skills and competencies to implement the activities in the classrooms. Moreover, through a pre- and post-test design, we observed an enhancement of protective cognitive factors, i.e., correct gambling knowledge, randomness understanding, and probabilistic reasoning, and a reduction of risk affective factors, i.e., superstitious thinking and economic positive gambling outcome expectation, as well as gambling-related cognitive distortions, in adolescents who attended the intervention. We also found a reduction of gambling frequency at the follow-up with respect to the baseline assessment. These results are overall in line with what previously found in some interventions with respect to specific risk and protective factors [11, 15, 16, 65–67]. Additionally, this is one of the few studies obtaining encouraging results in both cognitive and affective factors. In this regards, the Keen and colleagues' [8] systematic review about gambling prevention interventions for adolescents attest that the nineteen studies (out of the 69 papers assessed for eligibility) that conducted an evaluation of the intervention's effects were effective in improving cognitive outcomes such has beliefs, knowledge and attitudes, while it was harder to modify affective factors. Thus, this is a further confirmation of the adequacy of the conceptual change model [33] used as educational strategy in doing the activity in producing a change also of affective characteristics, as found in previous interventions that have applied this method [11, 64].

Importantly, in this study, we showed a not previously encountered result, as we found that adolescents reduced the prevalence of fallacious choices – and, conversely, they improved the normative choices – at the *Gambling Task* from the pre-test to the post-test. This is a very important result as it is likely to suggest that adolescents have learnt to be more adherent to their *mindware* resources – and less susceptible to *mindware contaminations* – when they are in front of risky decision-making situations, like that of a gambling activity. In this regard, we can hypothesize that the trained professional figures have been particularly able to manage the educational activities aimed at making the adolescents aware of the typical dual-characterization of the adolescent brain, with a less developed cognitive system and a particularly active socio-affective system, that makes them vulnerable to do risky decisions, especially when there are in hot contexts, i.e., situations in which socio-emotional features as peer pressure or the possibility to gain money, that function as activators of the socio-affective system [68, 69].

Another central issue concerns with the obtained reduced gambling frequency from the pre-test to the follow-up. As reported by Keen and colleagues [8], assessment of the behavioral changes reached in the gambling preventive interventions towards adolescents have been rarely conducted, with the majority of the studies that did not follow up with youth after a long

period of time. Thus, although research shows the difficulty of impacting on actual gambling behavior, our results suggest that the PRIZE program has been able in producing a transference of knowledge towards behavioral change. Moreover, in doing the behavioral change assessment, we confirmed the findings of the previous experimental study [11] that the program had no iatrogenic effects on gambling frequency. In this regard, we can notice that our follow-up measurement session was not conducted at 6 months, as other studies have done (e.g., 65), as doing it after 6 months would have implied the end of the school year and the passage to the following school year and therefore the possible loss of participants from the study. However, a follow-up at 3-4 months by the intervention is consistent with a lot of previous intervention studies (e.g., 14, 15, 67), and the obtained result is still encouraging and confirms the program's effectiveness, considering that one of the risks of creating a program aimed at preventing risky behavior is precisely the potentially harmful impact - primarily behavioral effects - in that it arouses curiosity in participants and encourages them to participate in risky behavior [70].

This finding further confirms the adequacy of broader and multidimensional approaches to ensure program effectiveness [71]. Such broader approach should be read both from a content point of view (i.e., we addressed both cognitive and affective factors) and by a delivery perspective (i.e., we used interactive games and activities, collective discussions after individual reflective exercises). More broadly, overall, this work clearly evidences that prevention programs delivered by specialized and trained staff are effective [72], and this highlights the importance of training prevention program providers and developing preventive initiatives with a solid theoretical foundation.

Additional findings regard the fact that once again we highlight that gambling behavior is widespread among adolescents [3]. In this regard, we have to consider that the landscape of gambling internationally has evolved at an unprecedented rate. While traditional land-based gambling continues to flourish, technological advances have enabled adolescents to wager from the comfort of their own homes [73]. In particular, during recent years, technological innovations and increased access to Internet mediated gambling and gaming have blurred the lines between monetary gambling and gaming activities [74, 75]. One particular aspect of this development is the integration of in-game purchases and gambling-like elements in video gaming with the so-called loot boxes [76]. Other factors that may have had a role in reinforcing adolescents' involvement in gambling is gambling advertisement [77, 78]. Indeed, gambling is typically advertised as a harmless form of entertainment and an enjoyable fun, leisure time activity [79, 80], while the harmful consequences of excessive gambling are generally framed as an issue of choice [81]. Thus, the underlying perceived message is that winning is easy, the chance of winning is high and gambling is an easy way to acquire money and wealth. Young people are exposed to such kind of messages, through pop-up ads on the Internet, newspapers, radio and TV, magazines.

Despite the highlighted strengths, this work has some limitations. Indeed, measurement scales of some variables considered to test the effectiveness of the training course was carried out with instruments created *ad hoc* and therefore, this could put limits on some findings obtained. Furthermore, the PRIZE program was implemented by numerous (although previously trained) operators, which means that the method of implementation was not completely uniform, especially considering their different initial vocation and competencies. Moreover, all the health professionals who carried out the PRIZE program were trained without a control group. Finally, due to the longitudinal nature of the intervention, we have to register a high attrition rate from the pre-test and the post-test. However, we controlled that there were only small in size differences in terms of gender and age between adolescents that completed the post-test and those who did not complete it and, more importantly, that the differences in

gambling characteristics between adolescents who completed the long-term evaluation and those who did not were very small in size. Future research should better evaluate the effects of the training course for the intervention providers by using a control group, and by employing more sophisticated assessment instruments. Additionally, as this work shows some particularly vulnerable groups, i.e. boys and adolescents with migration background, specific targeted preventive initiatives should be developed and evaluated with them. Indeed, secondary prevention is the most adequate to design initiatives for youth at-risk, in order to reduce the likelihood of developing severe problem gambling behaviours [71].

In sum, this work highlights the importance of delivering and assessing training prevention program providers to optimize the effectiveness of gambling intervention programs towards adolescents.

## Supporting information

**S1 File.**
(SAV)

## Acknowledgments

We thank all the intervention providers, the students who participated in PRIZE, their teachers, headmasters, and parents.

## Author Contributions

**Conceptualization:** Maria Anna Donati, Caterina Primi.

**Data curation:** Jessica Boncompagni.

**Formal analysis:** Maria Anna Donati.

**Funding acquisition:** Giuseppe Iraci Sareri, Sonia Ridolfi.

**Investigation:** Maria Anna Donati.

**Methodology:** Maria Anna Donati, Caterina Primi.

**Project administration:** Giuseppe Iraci Sareri, Sonia Ridolfi, Adriana Iozzi, Valentina Cocci, Alfiero Arena.

**Supervision:** Caterina Primi.

**Writing – original draft:** Maria Anna Donati, Jessica Boncompagni.

**Writing – review & editing:** Maria Anna Donati, Caterina Primi.

## References

1. Ferris J, Wynne H, & Single E. Measuring problem gambling in Canada: Draft final report for the interprovincial task force on problem gambling Ottawa. Ontario: Canadian Centre on Substance Abuse, 1998.
2. Volberg RA, Gupta R, Griffiths MD, Ólason DT, & Delfabbro P. An international perspective on youth gambling prevalence studies. In: Derevensky JL, Shek DTL, Merrick Jeditors. Youth gambling: The Hidden Addiction. Berlin, Boston: De Gruyter; 2011. pp. 21–56. https://doi.org/10.1515/9783110255690
3. Calado F, Alexandre J, & Griffiths MD. Prevalence of adolescent problem gambling: A systematic review of recent research. J Gambl Stud. 2017; 33(2), 397–424. https://doi.org/10.1007/s10899-016-9627-5 PMID: 27372832
4. www.statista.com

5. The ESPAD Group. ESPAD Report 2019. Results from the European School Project on Alcohol and Other Drugs. Available from: http://www.espad.org/espad-report-2019

6. Donati MA, Derevensky JL, Cipollini B, Leonardo LD, Sareri GI, & Primi C. Measuring gambling outcome expectancies in adolescents: testing the psychometric properties of a Modified Version of the Gambling Expectancy Questionnaire. J Gambl Stud. 2022; 1–22. 7. https://doi.org/10.1007/s10899-021-10029-y PMID: 33997939

7. Buja A, Mortali C, Mastrobattista L, Minutillo A, Pichini S, Genetti B, et al. Pathways connecting socio-economic variables, substance abuse and gambling behaviour: a cross-sectional study on a sample of Italian high-school students. BMJ Open. 2019; 9(11), e031737. https://doi.org/10.1136/bmjopen-2019-031737 PMID: 31719086

8. Keen B, Blaszczynski A, & Anjoul F. Systematic review of empirically evaluated school-based gambling education programs. J Gambl Stud. 2017; 33(1), 301–325. https://doi.org/10.1007/s10899-016-9641-7 PMID: 27566689

9. Ladouceur R, Goulet A, & Vitaro F. Prevention programmes for youth gambling: a review of the empirical evidence. Int Gambl Stud. 2013; 13(2), 141–159. https://doi.org/10.1080/14459795.2012.740496

10. Oh BC, Ong YJ, & Loo JM. A review of educational-based gambling prevention programs for adolescents. Asian J Gambl Issues Public Health. 2017; 7(1), 1–16. https://doi.org/10.1186/s40405-017-0021-8 PMID: 28316902

11. Donati MA, Chiesi F, Iozzi A, Manfredi A, Fagni F, & Primi C. Gambling-related distortions and problem gambling in adolescents: A model to explain mechanisms and develop interventions. Front Psychol, 2018; 8, 2243. https://doi.org/10.3389/fpsyg.2017.02243 PMID: 29354081

12. Lemaire J. De Lima S, & Patton D. It's your lucky day: Program evaluation. 2004. The Addictions Foundation of Manitoba. Available from Addictions Foundation of Manitoba website http://www.afm.mb.ca/pdf/Lucky%20Day%20Report, 202004.

13. Lupu IR & Lupu V. Gambling prevention program for teenagers. Cogn Behav Psychother, 2013; 13 (2a), 575–584.

14. Turner NE, Macdonald J, & Somerset M. Life skills, mathematical reasoning and critical thinking: A curriculum for the prevention of problem gambling. J Gambl Stud. 2008; 24(3), 367–380. https://doi.org/10.1007/s10899-007-9085-1 PMID: 18095146

15. Walther B, Hanewinkel R, & Morgenstern M. Short-term effects of a school-based program on gambling prevention in adolescents. J Adolesc Health. 2013; 52(5), 599–605. https://doi.org/10.1016/j.jadohealth.2012.11.009 PMID: 23375824

16. Calado F, Alexandre J, Rosenfeld L, Pereira R, & Griffiths MD. The efficacy of a gambling prevention program among high-school students. J Gambl Stud. 2020; 36(2), 573–595. https://doi.org/10.1007/s10899-019-09908-2 PMID: 31712967

17. Nation M, Crusto C, Wandersman A, Kumpfer KL, Seybolt D, Morrissey-Kane E, et al. What works in prevention: Principles of effective prevention programs. Am Psychol. 2003; 58(6–7), 449–456. https://doi.org/10.1037/0003-066x.58.6-7.449 PMID: 12971191

18. Ahmed N, Flisher AJ, Mathews C, Jansen S, Mukoma W, & Schaalma H. Process evaluation of the teacher training for an AIDS prevention programme. Health Educ Res. 2006; 21(5), 621–632. https://doi.org/10.1093/her/cyl031 PMID: 16740671

19. Schaalma HP, Abraham C, Gillmore MR, & Kok G. Sex education as health promotion: what does it take?. Arch Sex Behav. 2004; 33(3), 259–269. https://doi.org/10.1023/B:ASEB.0000026625.65171.1d PMID: 15129044

20. Stang JS, Story M, & Kalina B. School-based weight management services: perceptions and practices of school nurses and administrators. Am J Health Promot. 1997; 11(3), 183–185. https://doi.org/10.4278/0890-1171-11.3.183 PMID: 10165096

21. Hall G. & Hord S. Implementing change: Patterns, principles and potholes. Boston, MA: Allyn and Bacon. 2001.

22. Kumpfer KL & Alvarado R. Family-strengthening approaches for the prevention of youth problem behaviors. Am Psychol. 2003; 58(6-7), 457–465. https://doi.org/10.1037/0003-066X.58.6-7.457 PMID: 12971192

23. Flay BR, Biglan A, Boruch RF, Castro FG, Gottfredson D, Kellam S, et al. Standards of evidence: Criteria for efficacy, effectiveness and dissemination. Prev Sci. 2005; 6(3), 151–175. https://doi.org/10.1007/s11121-005-5553-y PMID: 16365954

24. Toplak M, Liu E, Macpherson R, Toneatto T, & Stanovich KE. The reasoning skills and thinking dispositions of problem gamblers: A dual-process taxonomy. J Behav Decis Mak. 2007; 20, 103–124. http://dx.doi.org/10.1002/bdm.544

25. Stanovich KE. Balance in psychological research: The dual process perspective. Behav Brain Sci. 2004; 27(3), 357–358. https://doi.org/10.1017/S0140525X0453008X

26. Raylu N & Oei TP. The Gambling Related Cognitions Scale (GRCS): Development, confirmatory factor validation and psychometric properties. Addiction. 2004; 99(6), 757–769. https://doi.org/10.1111/j.1360-0443.2004.00753.x PMID: 15139874

27. Estevez A, Jauregui P, Lopez-Gonzalez H, Mena-Moreno T, Lozano-Madrid M, Macia L, et al. The severity of gambling and gambling related cognitions as predictors of emotional regulation and coping strategies in adolescents. J Gambl Stud. 2021; 37(2), 483–495. https://doi.org/10.1007/s10899-020-09953-2 PMID: 32436155

28. Perkins DN. Outsmarting IQ: The Emerging Science of Learnable Intelligence. New York, NY: Free Press, 1995.

29. Delfabbro P, Lambos C, King D, & Puglies S. Knowledge and beliefs about gambling in Australian secondary school students and their implications for education strategies. J Gambl Stud. 2009; 25, 523–539. https://doi.org/10.1007/s10899-009-9141-0 PMID: 19653079

30. Ninness HAC & Ninness SK. Superstitious math performance: interactions between rule-governed and scheduled contingencies. Psychol Rec. 1998; 48, 45–62. https://doi.org/10.1007/BF03395258

31. Moore SM & Ohtsuka K. Beliefs about control over gambling among young people, and their relation to problem gambling. Psychol Addict Behav. 1999; 13(4), 339–347. https://doi.org/10.1037/0893-164X.13.4.339

32. West RF, Toplak ME, & Stanovich KE. Heuristics and biases as measures of critical thinking: associations with cognitive ability and thinking dispositions. J Educ Psychol. 2008; 100, 930–941. https://doi.org/10.1037/a0012842

33. Posner GJ, Strike KA, Hewson PW, & Gertzog WA. Accommodation of a scientific conception: Toward a theory of conceptual change. Sci Educ. 1982; 66(2), 211–227. https://doi.org/10.1002/sce.3730660207

34. Forsström D, Spångberg J, Petterson A, Brolund A, & Odeberg J. A systematic review of educational programs and consumer protection measures for gambling: an extension of previous reviews. Addict Res Theory. 2021; 29(5), 398–412. https://doi.org/10.1080/16066359.2020.1729753

35. Sogunro OA. Efficacy of role-playing pedagogy in training leaders: some reflections. J Manag Dev. 2004; 23(4), 355–371. https://doi.org/10.1108/02621710410529802

36. Donati MA, Beccari C, Biganzoli A, Tadini M, Capitanucci D, Smaniotto R, et al. Measuring gambling knowledge in adolescents: The construction of a new short scale for research and practice. Mental Health Addict Res. 2019; 4, 1–5. http://dx.doi.org/10.15761/MHAR.1000173

37. Primi C, Morsanyi K, Donati MA, Galli S, & Chiesi F. Measuring probabilistic reasoning: The construction of a new scale applying item response theory. J Behav Decis Mak. 2017; 30(4), 933–950. https://doi.org/10.1002/bdm.2011

38. Kahneman D, Lovallo D, &Sibony O. "The big idea. Before you make that big decision", Harvard Business Review, Vol. 89 No. 6, pp. 50–60. 2011. PMID: 21714386

39. Dufour J, Ladouceur R, & Giroux I. Training program on responsible gambling among video lottery employees. Int Gambl Stud. 2010; 10(1), 61–80. https://doi.org/10.1080/14459791003743037

40. LaPlante DA, Gray HM, LaBrie RA, Kleschinsky JH, & Shaffer HJ. Gaming industry employees' responses to responsible gambling training: A public health imperative. J Gambl Stud. 2012; 28(2), 171–191. https://doi.org/10.1007/s10899-011-9255-z PMID: 21656199

41. Memon MA, Salleh R, & Baharom MNR. The link between training satisfaction, work engagement and turnover intention. Eur J Train Dev. 2016; 40(6), 407–429.

42. Latif KF, Jan S, & Shaheen N. Association of Training Satisfaction with Employee Development aspect of Job Satisfaction. J Manag Sci. 2013; 7(1), 160–178.

43. Stajkovic AD, & Luthans F. Self-efficacy and work-related performance: A meta-analysis. Psychol Bull. 1998; 124(2), 240–261. https://doi.org/10.1037/0033-2909.124.2.240

44. Donati MA & Primi C. Psychometric properties of the Random Events Knowledge Test among Italian adolescents. 2021.

45. Turner NE, MacDonald J, Bartoshuk M, & Zangeneh M. Adolescent gambling behavior, attitudes, and gambling problems. Int J Ment Health Addict. 2008; 6, 223–237. http://dx.doi.1007/s11469-007-9117-1

46. Donati MA, Chiesi F, & Primi C. The role of probabilistic reasoning abilities on adolescent risk taking. Math Enthus. 2015; 12(1), 262–274.

47. Chiesi F, Donati MA, Papi C, & Primi C. Misurare il pensiero superstizioso nei bambini: validità e attendibilità della Superstitious Thinking Scale [Measuring superstitious thinking in children: validity and reliability of the Superstitious Thinking Scale]. *Età Evolutiva*. 2010; 97, 9–19.

48. Kokis JV, MacPherson R, Toplak ME, West RF, & Stanovich KE. Heuristic and analytic processing: age trends and associations with cognitive ability and cognitive styles. J Exp Child Psychol. 2002; 83, 26–52. https://doi.org/10.1016/s0022-0965(02)00121-2 PMID: 12379417
49. Gillespie MAM, Derevensky J, & Gupta R. Adolescent problem gambling: Developing a gambling expectancy instrument. J Gambl Issues. 2007; 19, 51–68. https://doi.org/10.4309/jgi.2007.19.3
50. Donati MA & Primi C. Psychometric properties of a modified form for adolescents of the Gambling Related Cognitions Scale. 2021.
51. Donati MA, Ancona F, Chiesi F, & Primi C. Psychometric properties of the Gambling Related Cognitions Scale (GRCS) in young Italian gamblers. Addict Behav. 2015; 45, 1–7. https://doi.org/10.1016/j.addbeh.2015.01.001 PMID: 25618794
52. Toneatto T. Cognitive psychopathology of problem gambling. Subst Use Misuse. 1999; 34(11), 1593–1604. https://doi.org/10.3109/10826089909039417 PMID: 10468110
53. Primi C, Donati MA, & Chiesi F. Gambling Behavior Scale for Adolescents. Scala per la Misura del Comportamento di Gioco D'azzardo Negli Adolescenti. Hogrefe Editore. 2015.
54. American Psychiatric Association. Diagnostic and statistical manual of mental disorders ( 5th ed.). American Psychiatric Association. 2013.
55. Ciccarelli M, Nigro G, D'Olimpio F, Griffiths MD, & Cosenza M. Mentalizing failures, emotional dysregulation, and cognitive distortions among adolescent problem gamblers. J Gambl Stud. 2021; 37(1), 283–298. https://doi.org/10.1007/s10899-020-09967-w PMID: 32720217
56. Dowling NA, Merkouris SS, Greenwood CJ, Oldenhof E, Toumbourou JW, & Youssef GJ. Early risk and protective factors for problem gambling: A systematic review and meta-analysis of longitudinal studies. Clin Psychol Rev. 2017; 51, 109–124. https://doi.org/10.1016/j.cpr.2016.10.008 PMID: 27855334
57. Wijesingha R, Leatherdale ST, Turner NE, & Elton-Marshall T. Factors associated with adolescent online and land-based gambling in Canada. Addict Res Theory. 2017; 25(6), 525–532. https://doi.org/10.1080/16066359.2017.1311874
58. Canale N, Griffiths MD, Vieno A, Siciliano V, & Molinaro S. Impact of Internet gambling on problem gambling among adolescents in Italy: Findings from a large-scale nationally representative survey. Comput Hum Behav. 2016; 57, 99–106. https://doi.org/10.1016/j.chb.2015.12.020
59. Elton-Marshall T, Leatherdale ST, & Turner NE. An examination of internet and land-based gambling among adolescents in three Canadian provinces: results from the youth gambling survey (YGS). BMC Public Health. 2016; 16(1), 1–10. https://doi.org/10.1186/s12889-016-2933-0 PMID: 26987439
60. Donati MA, Chiesi F, & Primi C. A model to explain at risk/problem gambling among male and female adolescents: Gender similarities and differences. J Adolesc. 2013; 36, 129–137. https://doi.org/10.1016/j.adolescence.2012.10.001 PMID: 23177386
61. Weidberg S, González-Roz A, Fernández-Hermida JR, Martínez-Loredo V, Grande-Gosende A, García-Pérez Á, et al. Gender differences among adolescent gamblers. Person Individ. 2018; 125, 38–43. https://doi.org/10.1016/j.paid.2017.12.031
62. Canale N, Vieno A, Griffiths MD, Borraccino A, Lazzeri G, Charrier L, et al. A large-scale national study of gambling severity among immigrant and non-immigrant adolescents: the role of the family. Addict Behav. 2017; 66, 125–131. https://doi.org/10.1016/j.addbeh.2016.11.020 PMID: 27930902
63. Donati MA, Primi C, Mazzarese M, Sanson F, & Leone L. Immigrant status and problem-gambling severity in adolescents: Evidence for moderation by sensation seeking. Addict Behav. 2020; 107, 106395. https://doi.org/10.1016/j.addbeh.2020.106395 PMID: 32199193
64. Donati MA, Primi C, & Chiesi F. Prevention of problematic gambling behavior among adolescents: Testing the efficacy of an integrative intervention. J Gambl Stud. 2014; 30(4), 803–818. https://doi.org/10.1007/s10899-013-9398-1 PMID: 23832756
65. Huic A, Kranzelic V, Hundric DD, & Ricijas N. Who really wins? Efficacy of a Croatian youth gambling prevention program. J Gambl Stud. 2017; 33(3), 1011–1033. https://doi.org/10.1007/s10899-017-9668-4 PMID: 28108811
66. Dodig Hundric D, Mandic S, & Ricijas N. Short-Term Effectiveness of the Youth Gambling Prevention Program "Who Really Wins?"—Results from the First National Implementation. Int J Environ. 2021; 18 (19), 10100. https://doi.org/10.3390/ijerph181910100 PMID: 34639404
67. Williams RJ, Wood RT, & Currie SR. Stacked deck: An effective, school-based program for the prevention of problem gambling. J Prim Prev. 2010; 31(3), 109–125. https://doi.org/10.1007/s10935-010-0212-x PMID: 20405219
68. Casey BJ, Galván A, & Somerville LH. Beyond simple models of adolescence to an integrated circuit-based account: A commentary. Dev Cogn Neurosci. 2016; 17, 128–130. https://doi.org/10.1016/j.dcn.2015.12.006 PMID: 26739434

**69.** Shulman EP, Smith AR, Silva K, Icenogle G, Duell N, Chein J, et al. The dual systems model: Review, reappraisal, and reaffirmation. Dev Cogn Neurosci. 2016; 17, 103–117. https://doi.org/10.1016/j.dcn.2015.12.010 PMID: 26774291

**70.** Werch CE & Owen DM. Iatrogenic effects of alcohol and drug prevention programs. J Stud Alcohol. 2002; 63(5), 581–590. https://doi.org/10.15288/jsa.2002.63.581 PMID: 12380855

**71.** Kourgiantakis T, Stark S, Lobo DSS, & Tepperman L. Parent problem gambling: A systematic review of prevention programs for children. J Gambl Issues. 2016; 33, 8–29. https://doi.org/10.4309/jgi.2016.33.2

**72.** Todirita IR & Lupu V. Gambling prevention program among children. J Gambl Stud. 2013; 29(1), 161–169. https://doi.org/10.1007/s10899-012-9293-1 PMID: 22314758

**73.** Derevensky JL & Gilbeau. Preventing adolescent gambling problems. In: Heinz A, Romanczuk-Seiferth N, Potenza MNeditors. Gambling Disorder (pp. 297–311). Berlin, Germany. Springer, Cham; 2019. pp. 297-311.

**74.** Gainsbury SM. Gaming-gambling convergence: Research, regulation, and reactions. Gaming Law Rev. 2019; 23(2), 80–83. https://doi.org/10.1089/glr2.2019.2323

**75.** Teichert T, Gainsbury SM, & Mühlbach C. Positioning of online gambling and gaming products from a consumer perspective: A blurring of perceived boundaries. Comput Hum Behav. 2017; 75, 757–765. https://doi.org/10.1016/j.chb.2017.06.025

**76.** Kristiansen S & Severin MC. Loot box engagement and problem gambling among adolescent gamers: Findings from a national survey. Addict Behav. 2020; 103, 106254. https://doi.org/10.1016/j.addbeh.2019.106254 PMID: 31881408

**77.** Binde P & Romild U. Self-reported negative infuence of gambling advertising in a Swedish population-based Sample. J Gambl Stud. 2019; 35(2), 709–724. https://doi.org/10.1007/s10899-018-9791-x PMID: 29980884

**78.** Parke A, Harris A, Parke J, Rigbye J, & Blaszczynski A. Responsible marketing and advertising in gambling: A critical review. J Gambl Business Economics, 2015; 8(3), 21–35. https://doi.org/10.5750/jgbe.v8i3.972

**79.** Derevensky J, Sklar A, Gupta R, & Messerlian C. An empirical study examining the impact of gambling advertisements on adolescent gambling attitudes and behaviors. Int J Ment Health Addict. 2010; 8(1), 21–34. https://doi.org/10.1007/s11469-009-9211-7

**80.** Pitt H, Thomas SL, & Bestman A. Initiation, infuence and impact: Adolescents and parents discuss the marketing of gambling products during Australian sporting matches. BMC Public Health. 2016; 16(1), 1–12. https://doi.org/10.1186/s12889-016-3610-z

**81.** Korn D, Gibbins R, & Azmier J. Framing public policy towards a public health paradigm for gambling. J Gambl Stud. 2003; 19(2), 235–256. https://doi.org/10.1023/a:1023685416816 PMID: 12766471
